# Ancient DNA Study in Medieval Europeans Shows an Association Between HLA-DRB1*03 and Paratyphoid Fever

**DOI:** 10.3389/fimmu.2021.691475

**Published:** 2021-07-15

**Authors:** Magdalena Haller, Joanna H. Bonczarowska, Dirk Rieger, Tobias L. Lenz, Almut Nebel, Ben Krause-Kyora

**Affiliations:** ^1^ Institute of Clinical Molecular Biology, Kiel University, Kiel, Germany; ^2^ Department of Archaeology, Hanseatic City of Lübeck Historic Monuments Protection Authority, Lübeck, Germany; ^3^ Research Group for Evolutionary Immunogenomics, Max Planck Institute for Evolutionary Biology, Plön, Germany; ^4^ Research Unit for Evolutionary Immunogenomics, Department of Biology, Universität Hamburg, Hamburg, Germany

**Keywords:** *Salmonella enterica* Paratyphi C, enteric fever, human leukocyte antigen, trade-off, antigen binding prediction

## Abstract

Outbreaks of infectious diseases repeatedly affected medieval Europe, leaving behind a large number of dead often inhumed in mass graves. Human remains interred in two burial pits from 14^th^ century CE Germany exhibited molecular evidence of *Salmonella enterica* Paratyphi C (*S.* Paratyphi C) infection. The pathogen is responsible for paratyphoid fever, which was likely the cause of death for the buried individuals. This finding presented the unique opportunity to conduct a paratyphoid fever association study in a European population. We focused on HLA-DRB1*03:01 that is a known risk allele for enteric fever in present-day South Asians. We generated HLA profiles for 29 medieval *S.* Paratyphi C cases and 24 contemporaneous controls and compared these to a modern German population. The frequency of the risk allele was higher in the medieval cases (29.6%) compared to the contemporaneous controls (13%; *p = 0.189*), albeit not significantly so, possibly because of small sample sizes. Indeed, in comparison with the modern controls (*n = 39,689*; 10.2%; *p = 0.005*) the frequency difference became statistically significant. This comparison also suggested a slight decrease in the allele’s prevalence between the medieval and modern controls. Up to now, this is the first study on the genetic predisposition to *Salmonella* infection in Europeans and the only association analysis on paratyphoid fever C. Functional investigation using computational binding prediction between HLA variants and *S.* Paratyphi and *S.* Typhi peptides supported a reduced recognition capacity of bacterial proteins by DRB1*03:01 relative to other common DRB1 variants. This pattern could potentially explain the disease association. Our results suggest a slightly reduced predisposition to paratyphoid fever in modern Europeans. The causative allele, however, is still common today, which can be explained by a trade-off, as DRB1*03:01 is protective against infectious respiratory diseases such as severe respiratory syndrome (SARS). It is thus possible that the allele also provided resistance to corona-like viruses in the past.

## Introduction

In Europe, infectious diseases were particularly common in the Middle Ages (1–6), occasionally reaching immense proportions and wreaking havoc across the whole continent. For instance, the infamous Black Death and subsequent plague outbreaks left behind millions of dead who, due to the scale of the pandemic, were often inhumed in mass burials (7). Studying past contagions is not only of interest from the historical perspective, but also important for our understanding of current and future epidemics. Excavation on the grounds of the medieval Heiligen-Geist-Hospital (HGH) in Lübeck, northern Germany, revealed the presence of two mass graves and two smaller burial pits (8–10). Molecular analysis of the human remains (1370 – 1400 CE) indicated that individuals buried in the pits were infected with *Salmonella enterica* subsp. *enterica* serovar Paratyphi C at the time of death (11). The pathogen is a causative agent of paratyphoid fever, which is a life-threatening illness transmitted through contaminated food and water (12). Nowadays *S.* Paratyphi C is virtually absent in Europe, however, it can be assumed that the pathogen’s prevalence used to be higher in the past. Discovery of *S.* Paratyphi C in medieval Germany (11) and Norway (13) suggests a change in the geographic distribution of *Salmonella enterica* species over time. It is thus possible that paratyphoid fever used to have a substantial impact on the health of Europeans at that time (11). As the pathogen’s genome is thought to have been stable for millennia (13), genetic factors of the host could have contributed to the disappearance of this infectious disease in modern Europe.

Apart from *S.* Paratyphi C, paratyphoid fever can be caused by two other *Salmonella* serovars (*S.* Paratyphi A and B). Clinically similar typhoid fever is caused by *Salmonella* Typhi and together with *S.* Paratyphi infections, the diseases are known under the umbrella term of enteric fever. Several genetic host factors have been previously associated with susceptibility to the disease (14–17). Among those factors is the polymorphic human leukocyte antigen (HLA) region. The high level of HLA variation together with a number of associations between HLA alleles and a variety of infectious diseases [e. g. (18–24)] suggests that pathogens have acted as a powerful selective pressure on the human genome as a result of host-pathogen co-evolution (25–27). Regarding enteric fever, the HLA alleles DRB1*03:01 and DRB1*04:05 were identified as risk and protective factors, respectively, in present-day Vietnamese and Nepalese (16, 17). However, these associations were only shown for *S.* Typhi and *S.* Paratyphi A infections; as of yet, there are no studies exploring the genetic predisposition to *S.* Paratyphi C infections in modern populations. Nowadays the disease is very rare in Europe and usually a result of migrations from other regions of the world (12, 28, 29). Thus, analysis of susceptibility to paratyphoid fever in European populations is only possible with an ancient DNA (aDNA) approach. In this study, we generated HLA genotype data for 53 skeletal remains from the HGH site and in particular examined the frequency of the DRB1*03:01 allele to assess whether variation at the DRB1 locus predisposed medieval Europeans to enteric infection.

## Materials and Methods

### Materials

The skeletal material analyzed in this study was excavated from four mass burials (contexts 4528, 4529, 4562 and 4571) located near the Heiligen-Geist-Hospital (“Hospital of the Holy Ghost”, HGH) in Lübeck, northern Germany (8–10). The graves were dug between 1340 and 1370 CE (11).

### DNA Extraction and Library Preparation

Seventy individuals from the HGH site were analyzed in this study [Tables S1, Table S3 (11) for details on samples]. We sampled almost exclusively teeth and used the whole specimens for aDNA extraction. The DNA was isolated with the BioRobot^®^ EZ1 Advanced applying a custom-made version of the Large-Volume-Protocol and the EZ1 DNA Investigator^®^ Kit (Qiagen) as described previously (11, 30). The DNA extracts were then converted into indexed partial Uracil-DNA Glycosylase (UDG)-treated libraries. The libraries were sequenced on an Illumina HiSeq 4000 platform. To reliably exclude contamination, all procedures were conducted in clean rooms specifically dedicated to ancient DNA analysis, and negative controls were included in both the extraction and library preparation steps. Furthermore, short tandem repeat profiles at seven loci were generated as an additional authentication criterion and to avoid double sampling Table S2 (11).

### HLA Targeted Capture and Typing

In-solution enrichment of the HLA regions was successful for 53 of the 70 samples analyzed (excavation context 4528, *n = 13*; 4529, *n = 11*; 4562, *n = 6*; 4571, *n = 23*). Genotyping was performed using the semi-automated HLA-typing pipeline TARGT (Targeted Analysis of sequencing Reads for GenoTyping), which was designed for the analysis of low-coverage sequences as found in aDNA extracts (31). The pipeline automatically identifies and sorts target-specific reads from sequence data. Subsequently, the sorted sequences were manually analyzed to determine the HLA alleles (HLA calling) in individual samples (31). In this study, the manual genotyping was done by three researchers independently, thus increasing the reliability of the results. In addition, we applied the algorithm OptiType (32), which is only available for class I genes, to verify class I allele calls obtained with the TARGT pipeline. The OptiType software (32) was used with default parameters. Only those samples that had alleles concordantly called by both methods were included in the results (**Table S1**).

### Computational Prediction of Peptide Binding by HLA

Full proteomes of *Salmonella enterica* Paratyphi C RKS4594 (accession NC_012125.1, France) and *S. enterica* Typhi CT18 (accession NC_003198.1, Vietnam) were used to predict the binding of 15mer peptides (967,163 and 1,201,560 possible 15mers from *S.* Paratyphi and *S.* Typhi, respectively) for all DRB1 variants with an allele frequency >1% in the present-day German population (AlleleFrequencies.net Database (AFND): Germany, Population 8, *n = 39,689*; 33). Peptide binding prediction was performed with the established algorithm NetMHCIIpan v4.0, using the default threshold for ‘strong binding’ (33). For each HLA-DRB1 variant, the total number of bound peptides overall as well as the total number of bacterial proteins ‘recognized’ (at least one peptide of a given protein predicted to be bound by the given HLA-DRB1 variant) were obtained.

### Statistical Analysis

Fisher’s exact test and odds ratio calculations were performed for the DRB*03 allele using IBM SPSS Statistics (Version 26) predictive analytics software. Power calculations on required sample sizes were calculated with the software G*Power, v3.1.9.2 (34). For DRB1*03:01, the power calculation showed that an association analysis was appropriate with our sample size. Assuming an OR of 4 (17), an allocation ratio of 10 and an allele frequency of 10.2% in modern controls (35), 28 alleles were required in cases to obtain an *a priori* power of 75% at a significance level of 0.05.

## Results

**Figure 1:**
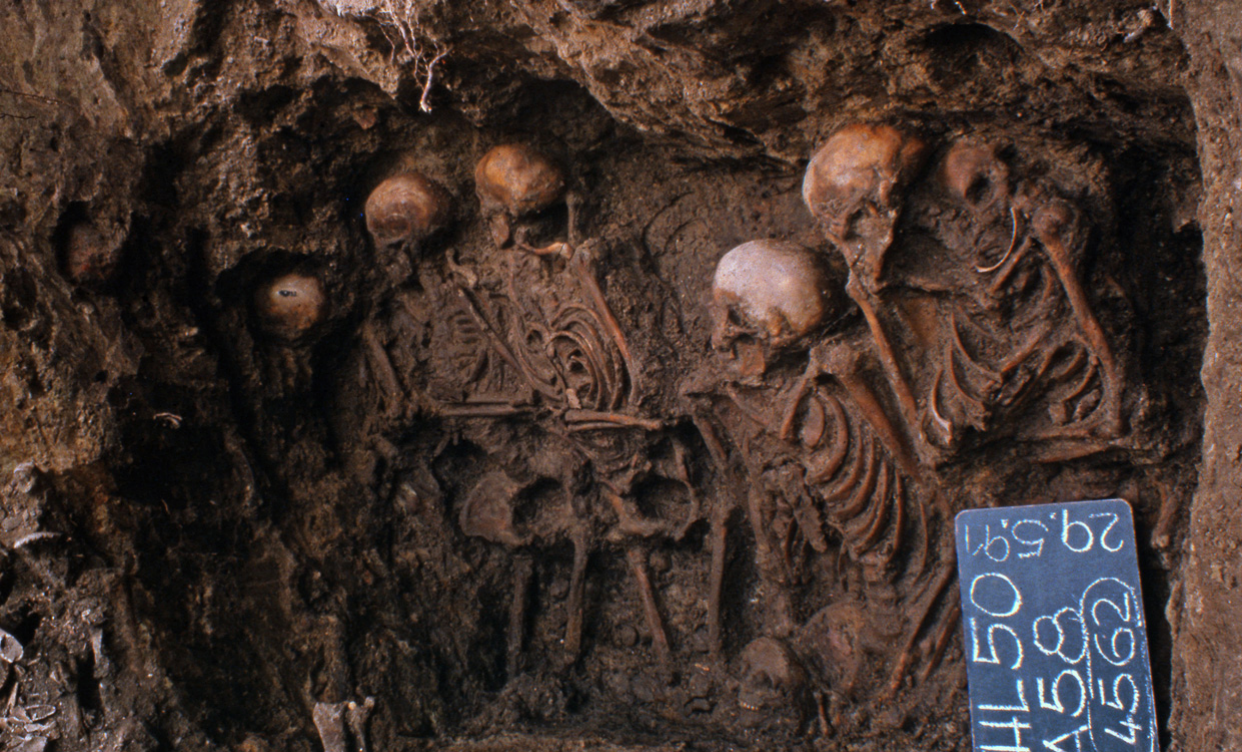
Heiligen-Geist-Hospital (“Holy-Ghost-Hospital”) archaeological context 4562 in which S. Paratyphi C-positive individuals were found.

In this study, we generated HLA genotype data from the skeletal remains of 53 individuals excavated from the HGH mass burial in Lübeck. The site consists of two mass graves (archaeological contexts 4528 and 4529) and two burial pits (4562 and 4571, **Figure 1**). Of the 70 metagenomic samples analyzed, 24 from 4528/4529 and 29 from 4562/4571 yielded HLA data after in-solution DNA enrichment. Genotyping of the HLA alleles was performed for the class I (HLA-A, -B and -C) and class II (HLA-DQB1 and -DRB1) loci using the TARGT pipeline which is well suited to the analysis of low-coverage sequences (31) (**Table S1**). Despite such a dedicated pipeline, the highly degraded nature of aDNA (36) poses a severe challenge to HLA typing so that allele calling at the preferred 2^nd^-field (4-digit) resolution is not always possible. Nevertheless, even at 1^st^-field level of resolution, HLA typing can be highly informative, as it distinguishes among major functional groups (‘supertypes’) of classical HLA alleles. For the same reason, it was also not always possible to call alleles at both haplotypes, so that the total number of called alleles was lower than 2N. The investigation here was performed following the strategy outlined for an association study with aDNA data (20). As the individuals buried in the pits (4562/4571) had been infected with *S.* Paratyphi C (11), they were considered cases in the association test (with 27 successfully called HLA-DRB1 alleles across the 29 individuals). The individuals buried in the mass graves (4528/4529) were used as contemporaneous controls (with 23 called HLA-DRB1 alleles across the 24 individuals), based on the fact that no molecular traces of the focal pathogen were observed in the remains (11). In a secondary analysis, allele frequencies in the historical cases were compared with a large cohort of modern individuals (AFND: Germany, Population 8, *n = 39,689*; 33).

As DRB1*04:05 is protective against enteric fever, its frequency would be expected to be lower relative to the modern controls (0.45%, Germany, AFND Population 8, *n = 39,689*; 33). Thus, DRB1*04:05 was not investigated due to the case sample being too small for a meaningful statistical analysis based on the power calculation. On the contrary, for the DRB1*03:01 risk allele, an association study was feasible making it the main focus of this investigation. Out of the 11 individuals with a DRB1*03 allele call at 1^st^-field resolution, calls at 2^nd^-field resolution were possible for four individuals who consistently showed DRB1*03:01. Among the known alleles of the DRB1*03 allele group, DRB1*03:01 is by far the most common in the modern German population (freq = 10.2%), with the next frequent allele being two orders of magnitude less frequent (DRB1*03:02, freq = 0.1%). It is therefore highly likely that the other seven individuals with a DRB1*03 call also carried the DRB1*03:01 allele, justifying an association analysis based on the DRB1*03 calls. Moreover, four individuals were possibly homozygous for the DRB1*03 type, given the available read depth and lack of other DRB1 sequences. However, a conservative approach was used here and these individuals were regarded as heterozygotes. It is thus likely that the obtained frequency is underestimated. The frequency of the DRB1*03 allele in the cases (29.6%) was considerably higher in comparison to the contemporaneous controls (13%, *p = 0.189*), albeit it did not reach statistical significance, likely owing to the small sample sizes for the medieval groups. In line with this suggestive association result, the comparison of medieval cases with modern individuals yielded a frequency difference that was highly significant (10.2%; *p = 0.005*, OR = 3.61) (**Table S1**). Interestingly, all four genotypes with a possible 2^nd^ field allele call of DRB1*03:01 also showed the alleles DQB1*02:01 or DQB1*02 at the neighboring HLA-DQB1 locus (**Table S1**), which could be an indication of genetic linkage between DRB1*03:01 and DQB1*02:01, a common haplotype in present-day populations (37).

**Figure 2:**
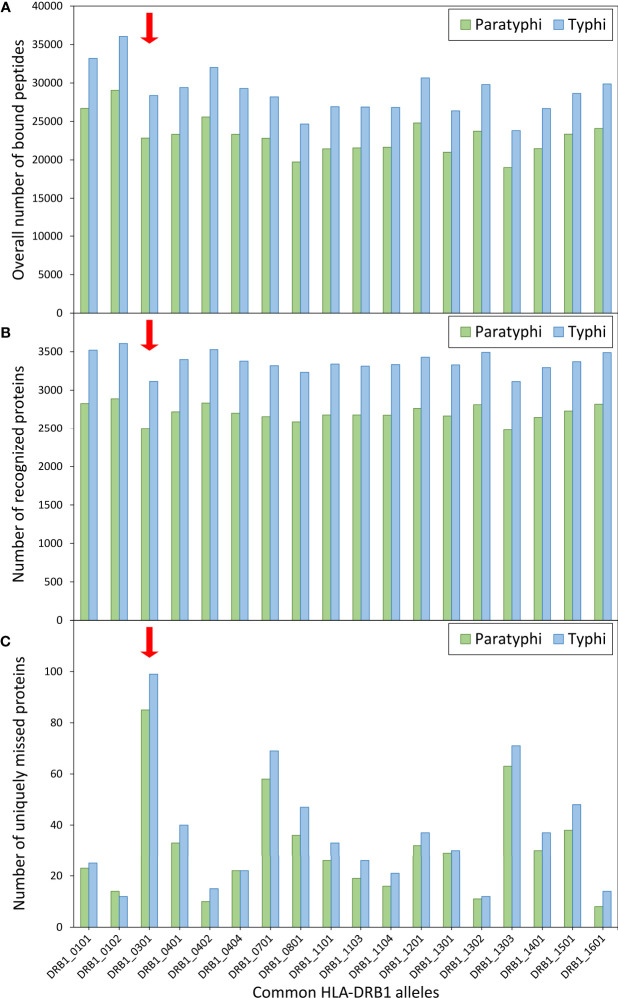
Computationally predicted binding of common HLA-DRB1 variants to bacterial peptides. Binding prediction was performed for all HLA-DRB1 variants with allele frequency >1% in present-day Germany (n=18), using the full proteomes of both Salmonella enterica Paratyphi C (green bars) and S. Typhi (blue bars). For each DRB1 variant, **(A)** the overall number of bound peptides, **(B)** the total number of ‘recognized’ proteins (at least one peptide bound by a given DRB1 variant), and **(C)** the number of uniquely ‘missed’ proteins (recognized by all other variants) are shown.

Given the role of HLA molecules in peptide presentation for antigen-specific immunity, the binding of *S.* Paratyphi and *S.* Typhi peptides to common HLA-DRB1 variants was also explored, including the risk variant DRB1*03:01. Using computational peptide binding prediction, no particular difference in the total number of bound bacterial peptides was observed between the risk variant and the other DRB1 variants (**Figure 2A**). However, the number of ‘recognized’ bacterial proteins (defined as proteins of which at least one peptide was bound by a given HLA variant) was among the lowest for DRB1*03:01 compared to the other HLA variants (for both *S.* Paratyphi C and *S.* Typhi; **Figure 2B**). This peculiarity in peptide binding of DRB1*03:01 became particularly evident when the number of proteins that were uniquely missed by each DRB1 variant (i.e., were predicted to be recognized by all other DRB1 variants but not the given one) were quantified. DRB1*03:01 missed 85 *S*. Paratyphi proteins that were recognized by all other DRB1 variants, more than thrice the number compared to the other DRB1 variants (median = 26 ± 16 SD, **Figure 2C**). A similar effect was observed for *S.* Typhi (**Figure 2C**).

## Discussion

In this study, variation at the HLA-DRB1 locus was investigated in the context of genetic susceptibility to *S.* Paratyphi C infection in human remains from Germany dating to the 14^th^ century CE. The DRB1*03:01 allele has been associated with an increased risk of enteric fever in modern Vietnamese and Nepalese populations (in this case caused by *S.* Typhi and *S.* Paratyphi A only) (16, 17). Up to date, no association study with an *S.* Paratyphi C infection has been performed in present-day populations, as it is relatively rare (38). However, there is evidence suggesting a high prevalence of this infectious disease in the Middle Ages (11, 13, 39).

HLA typing was successfully performed for 53 individuals excavated from four archaeological contexts: two mass graves (*n = 24*) and two smaller pits (*n = 29*). Individuals buried in the pits were found to exhibit genetic traces of infection with *S.* Paratyphi C, likely representing the cause of death. The larger mass graves were stratigraphically older and no pathogen DNA was detected in the remains inhumed there (11). It has been previously hypothesized that individuals in the mass graves were victims of plague (9, 40). However, no evidence has been found to support these claims (11). In addition, to the best of our knowledge, no link between HLA variation and plague susceptibility has been reported in human association studies. Thus, even if plague was responsible for the death of the individuals in the mass graves, the disease was not expected to influence the frequencies of the examined allele. Based on these assumptions, the individuals from the mass graves were treated as contemporaneous controls for the medieval paratyphoid fever cases buried in the small pits. The cases were also compared to a representative modern German population from the AlleleFrequencies.net Database (35). As genetic continuity between the HGH Lübeck population and present-day northern Europeans was shown previously (11), it allows for such an analysis to be carried out. Furthermore, kinship was not detected among the medieval individuals (11). Our results indicate that the frequency of DRB1*03 was significantly higher in the medieval paratyphoid fever cases in comparison to modern controls (*p = 0.005*). The obtained odds ratio of 3.6 is comparable to that in previous reports for enteric fever in modern South Asian populations (16, 17). The DRB1*03 frequency in the cases (29.6%) was also substantially higher relative to medieval controls (13%). Although calls at 2^nd^-field resolution (i.e., DRB1*03:01) were only possible for four individuals, all DRB1*03 calls were interpreted as DRB1*03:01, since this allele is by far the most common within the DRB1*03 group in Germans today (35).

This is the first report of an allelic association with an *S.* Paratyphi C infection. However, due to the relatively small number of specimens available for the present study, an inherent limitation of aDNA studies, this finding should be confirmed in a larger sample comprised of Far Eastern populations in which the disease is still common today (12). Nevertheless, as paratyphoid fever is no longer endemic in Europe, aDNA analysis is the only approach that allows us to explore whether DRB1*03:01 is involved in genetic predisposition to an *S.* Paratyphi C infection in Europeans.

The functional follow-up analysis, using a computational algorithm for prediction of binding between HLA variants and bacterial peptides, revealed an interesting pattern for the HLA-DRB1 risk variant that could potentially explain its association with susceptibility. The variant DRB1*03:01 was predicted to miss recognition (i.e., fail to bind at least one peptide) of the largest number of bacterial proteins that were recognized by all other common DRB1 variants. However, it should be noted that the number of missed proteins was small compared to the total number of proteins in the bacterial proteomes. It thus remains to be investigated whether this peculiar binding pattern of DRB1*03:01 would significantly affect susceptibility of its carriers.

The decreased frequency of the HLA risk allele in the modern population (10.2%) relative to the medieval controls (13%) might indicate slight negative selection. The limited available sample size for ancient remains for both cases and contemporaneous controls prevents any strong conclusion about the potential selective effect of this association. However, in view of the negative effect of DRB1*03:01, it is tempting to speculate about a trade-off that might explain as to why this allele is still present at such a high frequency today. Interestingly, the allele was shown to be protective against severe acute respiratory syndrome (SARS) infection in the modern Chinese population (41–43). Although currently there is no data confirming this association for present-day Europeans, it is possible that ancient epidemics of paratyphoid fever affected the level of genetic resistance against SARS, predisposing present-day Europeans to an infection with the causative coronavirus. There is further evidence linking DRB1*03:01 with various respiratory diseases such as resistance to tuberculosis (44). It is therefore conceivable that this allele was protective against corona-like viruses or other infectious agents in the past. In addition, it has been suggested that DRB1*03:01 provides protection against allergy to animal-derived proteins (dairy and eggs) (45, 46). Recently, we discovered that the allele was absent in a Neolithic population of 23 individuals (47). This observation is striking when considered in the context of dietary changes that were introduced with the Neolithic agricultural transition. All these factors and the notable presence of DRB1*03:01 in the current gene pool indicate that this HLA allele has been subject to balancing selection. DRB1*03:01 is a risk factor for type I diabetes, autoimmune hepatitis, multiple sclerosis, celiac disease, sarcoidosis, Grave’s disease, systemic lupus erythematosus and Sjörgen’s syndrome in modern populations across the globe (48–55). Due to the relatively low number of pathogens in the modern environment, negative effects of this HLA allele might now predominate, contributing to the increasing prevalence of inflammatory and autoimmune diseases today.

## Data Availability Statement

The datasets generated for this study can be found in the European Nucleotide Archive under the accession number PRJEB42927.

## Ethics Statement

Ethical review and approval were not required for the study on human participants in accordance with the local legislation and institutional requirements.

## Author Contributions

BK-K conceived and designed the study. MH generated aDNA data. MH, JB, and TL analyzed the data. MH, JB, TL, AN, and BK-K interpreted the results. DR provided data and interpretation on the archaeological material. JB, MH, TL, AN, and BK-K wrote the manuscript. All authors contributed to the article and approved the submitted version.

## Funding

This study was supported by the Deutsche Forschungsgemeinschaft (DFG, German Research Foundation) through the projects 2901391021 (CRC 1266), 437857095 and Germany’s Excellence Strategy EXC2167-390884018. JB was funded by the International Max Planck Research School for Evolutionary Biology.

## Conflict of Interest

The authors declare that the research was conducted in the absence of any commercial or financial relationships that could be construed as a potential conflict of interest.
